# Data bias

**DOI:** 10.1186/s13059-021-02278-2

**Published:** 2021-02-10

**Authors:** Teppo Felin, Jan Koenderink, Joachim I. Krueger, Denis Noble, George F. R. Ellis

**Affiliations:** 1 Saïd Business School, University of Oxford, Oxford, UK; 2 Department of Physics, Delft University of Technology, Delft, Netherlands; 3 Department of Experimental Psychology, University of Leuven, Leuven, Belgium; 4 Department of Cognitive, Linguistic and Psychological Sciences, Brown University, Providence, USA; 5 Department of Physiology, Anatomy and Genetics, University of Oxford, Oxford, UK; 6 Department of Mathematics, University of Cape Town, Cape Town, South Africa

Data is critical to science. But data itself is passive and inert. Data is not meaningful until it encounters an active, problem-solving observer. And in science, data gains relevance and becomes data in response to human questions, hypotheses, and theories.

In their response, Yanai and Lercher (Y&L) mis-specify the role of data and hypotheses in science (Yanai I, Lercher M.: The data-hypothesis conversation, forthcoming). Their insistence on the hypothesis-free exploration of data in scientific discovery is problematic. On the surface, they are right: data matters. And, as they note, the scientific process can indeed be convoluted, as data and hypotheses are tightly linked. But Y&L’s arguments suffer from a common bias where data is somehow seen as independent of hypothesis and theory. We respond to Y&L by revisiting their central points and examples.

## Data and observation is theory-dependent

Y&L argue that we “cling on to the notion that humanity is endowed with the wisdom to intuit new insights by philosophizing, *independent* of data and observations.” So, where exactly do new ideas and hypotheses come from, if not from data (as Y&L argue)?

Data does not have any qualities—whether important, surprising, or funny—without some kind of hypothesis or theory. There is nothing inherently meaningful or interesting about an apple falling, or any other data point, without a hypothesis or theory. It is only when the apple’s fall is met by a question and hypothesis that it takes on meaning. The very idea of analyzing an apple’s fall—the process of selecting that fall as data to be considered (and relating it to the moon!)—illustrates the central role of hypothesis in scientific discovery.

Now, perhaps Y&L agree that data is meaningless without a theory. After all, they soften their original “a hypothesis is a liability”-argument by allowing that various forms of “background” are important, even in hypothesis-free data exploration and scientific discovery. They refer to the importance of “theoretical background,” “mental background,” and “conceptual background.” This accumulated background, which allows scientists to build on the work of their predecessors, is undoubtedly important. And as Y&L further suggest, the scientific process is recursive, indeed, a conversation between data and theory.

However, this does not mean that scientific discovery and progress are deterministic or inevitable, where “each new question or hypothesis [is] triggered by the analysis of an earlier dataset” (Y&L). This cycle is not automatic. Science is not an all-seeing eye that is observer-independent [1]. It is necessarily punctuated by the human generative capacity to conjecture and hypothesize. It is conjecture, hypothesis and theory—rather than hypothesis-free exploration of data—that allow us to see something in a new way. Without this generative capacity, it is hard to fathom how we could know anything at all. As noted by the philosopher Charles Peirce, “man’s mind has a natural adaptation to imagining correct theories of some kinds…If man had not the gift of a mind adapted to his requirements, he could not have acquired any knowledge” [2].

Hypotheses can of course lead scientists astray, a point Y&L emphasize. We agree. But there is no meaningful, hypothesis-free alternative. The alternative to a bad or blinding hypothesis is a new or better one. Data, empirical findings, and obvious facts can also lead scientists astray. All observation is necessarily hypothesis-laden, no matter how informal these hypotheses might be. There is nothing inherent about data that tells us what to hypothesize. Again, data does not speak for itself. That is our point in focusing on the gorilla experiment, which appears to offer evidence of so-called human blindness. It similarly looks like the sun orbits the earth. But appearances and associated data can be deceiving. Therefore, a hypothesis tells us what data to look for, what experiments to construct, and how to interpret findings.

## The heart of the matter

To illustrate their points about the importance of data in science, Y&L focus on the research of one of us (Denis Noble and his team). We are surprised by Y&L’s analysis of “the surprising heart revisited”-article (Noble D.: The surprising heart revisited: an early history of the funny current with modern lessons, forthcoming), since their points are the opposite of what the experiments and analysis showed. Consider their question: “Had this ‘funnyness’ of the data been part of a pre-experimental hypothesis?” Y&L’s answer is “no.” However, the answer of the authors who performed the original heart studies is “yes.”

To understand this answer, we need to know that the context was a controversy on the mechanism of heart rhythm. The prevailing theory at the time was McAllister-Noble-Tsien’s (1975) model for rhythm in the conducting system. In 1979, the team extended the model to the natural rhythm generator, the sinus node. They found a channel that behaved almost exactly as predicted with regard to gating kinetics and voltage range. But it did not reverse on hyperpolarization. The funny nature of the current traces was strange *only within the context of that model*.

There is nothing intrinsically funny about data showing a current that continues to increase on hyperpolarization. There are many ion channels that do that. There would have been no funnyness at all had there not been a theory within which it seemed funny. Contrary to the claim that “it [the MNT theory] was not what these scientists had set out to test,” electrophysiologists would be lost in a surfeit of unexplained data if they did not have Nernst potential theory, Hodgkin-Huxley channel theory, and many other theoretical bases of electrophysiology, to guide them. They would not even have known why they might expect to find a reversal potential.

The misunderstanding is even deeper when Y&L imagine that “Noble and DiFrancesco finally had what they needed to build a model,” as though that was all they were interested in doing. As made clear in “the surprising heart revisited”-article, the theory DiFrancesco and Noble developed enabled a separation between misleading (the reversal potential) and non-misleading (the gating kinetics) data. Later experimental work had to rely on that distinction, *even using data that could not itself reveal the distinction*. That separation also relied on the perturbation theory analysis provided by DiFrancesco and Noble [3].

The distinction between misleading and non-misleading data is critical. This is shown by the fact that the theory gave a causal explanation for a phenomenon that would otherwise be just another potentially misleading association score. The theory explained why a channel that may normally conduct the largest depolarizing current in pacemaker rhythm can be blocked by an HCN blocker, ivabradine. *There was no hypothesis-independent way to see this*. The low association score would not have revealed its causal role because the association data alone does not reveal causation. In any case, the association score would be only around 15%, even when the causal role is up to 80%. This problem is of general importance in interpreting genome-wide association studies as well. Association scores do not reveal causation. It requires a link-up between the association data and physiological causation to achieve that goal [4]. Otherwise we are left puzzled by the fact that most gene association studies show remarkably low associations [5]. Interpreted with causal physiological analysis, those low scores are apparent, not real, but data alone will not show that.

In short, Y&L’s interpretation of the funny heart current represents a misreading of the data-theory relation. The primary driver of progress in this setting was offered by the hypotheses and theories, rather than the data.

## Top-down, organism-specific mechanisms

Y&L’s emphasis on data implicitly suggests a form of scientific reductionism and organism-independence. The increased availability of low-level data—like the genomes of various species—and widespread access to computational tools, underlie the overconfidence in data analysis and hypothesis-free scientific discovery. Data-focused approaches encourage reductionist forms of scientific investigation, often ignoring the role of top-down, organism- and observer-specific factors in science.

This form of reductionism is evident in one of Y&L’s previous editorials in *Genome Biology.* In this piece, they celebrate the book *The Selfish Gene* and argue:

“Most importantly, Dawkins demonstrated with the utmost lucidity that we had biology upside down: evolution—and hence biology—is not concerned with the organism, but with the genes that survive unscathed through the eons by jumping from body to body.” [6].

Perhaps life is genes-all-the-way-down for some geneticists. But that type of reductionism is problematic from a broader biological (and scientific) point of view. No gene survives unscathed, as Y&L would have it. Genes do not live independent of the organism. DNA is in fact coddled by the organism as it corrects its hundreds and thousands of errors each time it is copied and reorganizes the genome when it needs to find new solutions to keep evolving. In short, the focus on genes alone and organism-independence is misplaced. From our perspective, the above represents a “gloomy and discouraging view on account of the apparent passivity of the organism in the process of evolution.” [7].

We worry that the wealth of access to lower-level data (like genetic information) and powerful computational tools is leading researchers to be overly focused on low-level data at the expense of the exploration of various top-down mechanisms. We should celebrate the contribution that disparate levels and units of analysis and associated disciplines can make to our understanding of life [8]. The key question here is whether or not data at multiple levels is taken into account, or only data at lower levels. Selection does not take place at the level of the gene. It depends on emergent properties and ecological contexts [9]. Any advocacy for an organism-independent science of biology—as Y&L suggest above—or more general reductionist science are highly problematic. Efforts to understand evolution should be just as much concerned with top-down organism-specific and environmental factors (physiology, epigenetics) as bottom-up genetic ones. This is the reason we emphasized both bottom-up and top-down mechanisms in our original article.

A particular top-down influence we have sought to emphasize is the role that human conjectures, predictions, hypotheses, and theories play in science and our understanding of the world. Humans have a unique capacity for scientific investigation, for making generative predictions and conjectures. But in an important sense, as shown by comparative biologists, all life is engaged in a form of problem-solving and probing when searching and exploring its environment. This cannot be reduced to genes or any other form of lower-level data. Evolution is also directed by organism-specific factors and conjectures. Genes undoubtedly play a role, as does environmental selection. But the organism-specific contributions also deserve attention.
